# Detection of the Onset of Ischemia and Carcinogenesis by Hypoxia-Inducible Transcription Factor-Based *In Vivo* Bioluminescence Imaging

**DOI:** 10.1371/journal.pone.0026640

**Published:** 2011-11-10

**Authors:** Tetsuya Kadonosono, Takahiro Kuchimaru, Shuichi Yamada, Yumi Takahashi, Atsushi Murakami, Taeko Tani, Hitomi Watanabe, Tomoharu Tanaka, Kiichi Hirota, Masahiro Inoue, Tetsuya Tsukamoto, Takeshi Toyoda, Koji Urano, Kazuhiko Machida, Tomoo Eto, Tomoyuki Ogura, Hideki Tsutsumi, Mamoru Ito, Masahiro Hiraoka, Gen Kondoh, Shinae Kizaka-Kondoh

**Affiliations:** 1 Department of Biomolecular Engineering, Tokyo Institute of Technology Graduate School of Bioscience and Biotechnology, Nagatsuta-cho, Midori-ku, Yokohama, Japan; 2 Animal Research Laboratory, Bioscience Research and Education Center, Akita University, Akita, Japan; 3 Department of Radiation Oncology and Image-applied Therapy, Graduate School of Medicine, Kyoto, Japan; 4 Laboratory of Animal Experiments for Regeneration, Institute for Frontier Medical Sciences, Kyoto, Japan; 5 Department of Anesthesia, Kyoto University, Shogoin-Kawahara-cho, Sakyo-ku, Kyoto, Japan; 6 Department of Biochemistry, Osaka Medical Center for Cancer and Cardiovascular Disease, Nakamichi, Higashinari-ku, Osaka, Japan; 7 Division of Oncological Pathology, Aichi Cancer Center Research Institute, Nagoya, Japan; 8 Central Institute for Experimental Animals, Nogawa, Miyamae, Kawasaki, Japan; The University of Kansas Medical Center, United States of America

## Abstract

An animal model for the early detection of common fatal diseases such as ischemic diseases and cancer is desirable for the development of new drugs and treatment strategies. Hypoxia-inducible factor 1 (HIF-1) is a transcription factor that regulates oxygen homeostasis and plays key roles in a number of diseases, including cancer. Here, we established transgenic (Tg) mice that carry *HRE/ODD-luciferase* (*HOL*) gene, which generates bioluminescence in an HIF-1-dependent manner and was successfully used in this study to monitor HIF-1 activity in ischemic tissues. To monitor carcinogenesis *in vivo*, we mated HOL mice with rasH2 Tg mice, which are highly sensitive to carcinogens and are used for short-term carcinogenicity assessments. After rasH2-HOL Tg mice were treated with *N*-methyl-*N*-nitrosourea, bioluminescence was detected noninvasively as early as 9 weeks in tissues that contained papillomas and malignant lesions. These results suggest that the Tg mouse lines we established hold significant potential for monitoring the early onset of both ischemia and carcinogenesis and that these lines will be useful for screening chemicals for carcinogenic potential.

## Introduction

Hypoxia-inducible factors (HIFs) are transcription factors essential for cellular adaptation to hypoxia. More than 100 putative HIF direct target genes have been identified, and many of these are involved in adaptive processes to hypoxia [1]. HIF-1, the most abundant and ubiquitously expressed HIF, is composed of a hypoxia-inducible α subunit (HIF-1α) and a constitutively expressed β subunit (HIF-1β) [1]. Under well-oxygenated conditions, HIF-1α is hydroxylated by members of the prolyl hydroxylase domain (PHD) family, which target highly conserved prolyl residues (Pro402 and Pro564) located in the HIF-1α oxygen-dependent degradation (ODD) domain [1]–[4]. Trans-4-prolyl-hydroxylation of HIF-1α enables it to be recognized by the von Hippel-Lindau tumor suppressor protein (pVHL), a component of ubiquitin ligase complexes [3], [4]. As a result, HIF-1α is polyubiquitinated and undergoes proteasomal degradation [1]–[4]. Hypoxia suppresses the rate of HIF-1α hydroxylation, allowing HIF-1α to accumulate and translocate to the nucleus, where it heterodimerizes with HIF-1β to form an active transcription factor [1].

In addition to its oxygen-dependent, post-translational regulation, HIF-1α is regulated at various other levels, including transcription, translation, and PHD-independent protein degradation. In cancer cells, for example, insulin and certain growth factors are known to increase HIF-1α protein synthesis in a 59 untranslated region-dependent manner via the activation of the phosphatidylinositol 3-kinase/Akt pathway and mTOR signaling [5], [6]. Moreover, HIF-1α protein synthesis has been reported to be regulated by the RNA-binding proteins HuR and PTB and by the stress-induced phosphorylation of eIF2a [7]–[9]. There is also evidence for pathways that control HIF-1α stability in an oxygen-independent manner. For instance, Hsp90 inhibitors and the transcription factor FOXO-4 have been reported to induce the degradation of HIF-1α in a pVHL-independent manner [10]–[13]. Furthermore, genetic changes in oncogenes such as *raf* and *p53* have been shown to increase HIF-1α expression [1], [14]–[17].

In cancers, a number of the genes induced by HIF-1 are critically involved in processes such as immortalization, cellular differentiation, genetic instability, vascularization, metabolic reprogramming, autocrine growth factor signaling, invasion/metastasis, and resistance to treatment [1], [2], [14]. Therefore, HIF activity is potentially an excellent candidate for use as a marker of cancer formation. Recently we constructed an *HRE/ODD-luciferase* (*HOL*) vector [18] consisting of a HIF-1-dependent promoter and a cDNA encoding firefly luciferase fused to the sequence for the ODD domain (548–603) of human HIF-1α [19]. As a result of the PHD regulation of HIF through the ODD domain, the *in vivo* half-life of luciferase was shortened from ∼5 h to less than 17 min in the presence of a normal partial pressure of oxygen. This shortened half-life resulted in the avoidance of the prolonged accumulation of luciferase protein in cells lacking HIF-1 activity and thereby allowed a more precise detection of HIF-1 activity.

Whole-body bioluminescent imaging is a highly versatile and powerful tool for obtaining biological information from small animals in a noninvasive manner [20]. Bioluminescent imaging relies on the activity of enzymes that convert unique substrates into light and have enabled the monitoring of transgene expression in genetically engineered mice [21]. Bioluminescence at the emission wavelength of firefly luciferase (560 nm) can be imaged as deep as several centimeters within the tissue, which allows a resolution to at least the organ level.

In this study, we have constructed HOL Tg mice, which generate bioluminescence in a HIF-1-dependent manner. For monitoring tumor formation *in vivo*, HOL Tg mice were mated with rasH2 Tg mice [22], which show a higher sensitivity to carcinogens than conventional mice [23] and have been used for short-term carcinogenicity assessments as an alternative to the traditional 2-year mice bioassays [24]. bioluminescence was noninvasively detected as early as 9 weeks after treatment with *N*-methyl-*N*-nitrosourea (MNU), and the tissues with the bioluminescence contained papillomas and carcinomas. Overall, the results demonstrated that the Tg mice we developed can be used as mouse models for *in vivo* bioimaging in order to study the onset and progression of ischemic diseases and cancers.

## Results

### Development and characterization of Tg mice for monitoring HIF-1 activity

**Figure 1:**
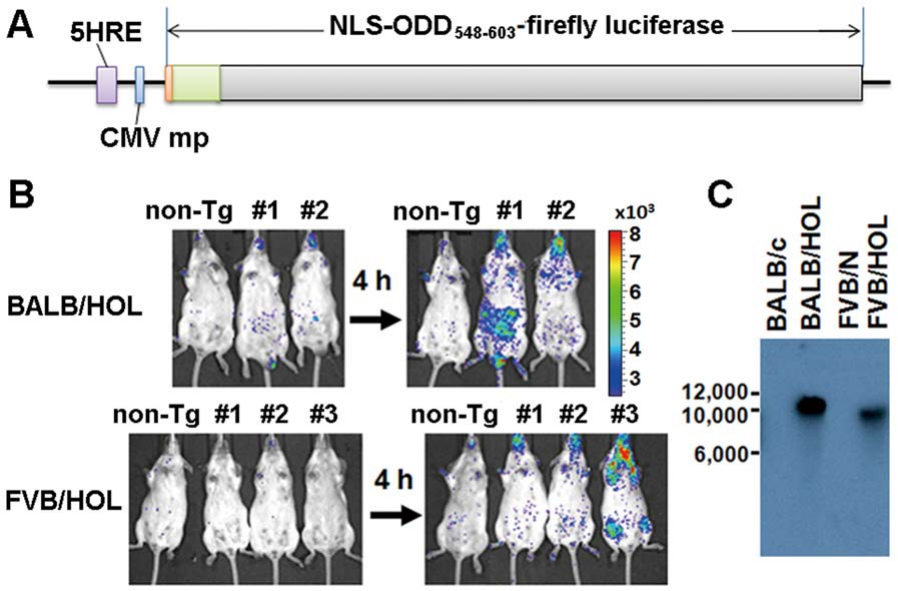
Generation and characterization of HOL transgenic mice. (A) Schematic representation of HRE/ODD-luciferase transgene. The promoter contains 5 tandem repeats of the hypoxia responsive element (HRE) of human vascular endothelial growth factor gene. The reporter gene encodes a fusion protein consisting of the nuclear localization signal (NLS) of the simian vacuolating virus 40 T-antigen, the oxygen-dependent degradation domain (ODD) of human HIF-1α (548–603) and the firefly luciferase. (B) FVB/HOL and BALB/HOL mice were intraperitoneally injected with 3,4,5-trihydroxybenzoic acid propyl ester (PG). The bioluminescence imaging was performed 4 h after PG injection. The experiment was repeated three times, and the representative data are shown. (C) Southern blot analysis of the *Pst*I-digested genome of the BALB/c, BALB/HOL, FVB/N, and FVB/HOL mice. The blot was probed with the reporter cDNA fragment of the transgene.

To noninvasively monitor HIF-1 activity, we generated several HOL Tg mouse lines (Figure 1A) in the BALB/c and FVB/N backgrounds (BALB/HOL and FVB/HOL, respectively). The *HOL* transgene expresses the ODD-luciferase fusion protein under the HIF-1-dependent promoter HRE and therefore generates bioluminescence in HIF-1 active cells. Moreover, the ODD-fusion protein is degraded quickly in HIF-inactive cells [18], [25], allowing real-time monitoring of HIF-1 activity in mice. Peritoneal injections with the antioxidant reagent propyl gallate (PG) were used to select for mouse lines with good induction of the bioluminescence signal (Figure 1B) since PG inhibits HIF prolyl hydroxylase and effectively stabilizes HIF-1 [26]. PG treatment can enable a significant HIF-1-induced bioluminescence signal to be observed over background bioluminescence (Figure S1). Among the different lines, line 1 of BALB/HOL Tg mice and line 3 of FVB/HOL Tg mice showed reliable expression of the transgene after PG treatment. Both lines were each found to carry a single copy of the transgene (Figure 1C). FVB/HOL line 3 was found to carry the transgene in chromosome 19, the smallest of the chromosomes (Figure S2). Since this line also showed a sharp turning on/off of *HOL* gene expression, we primarily used this line in the subsequent studies.

### Luciferase reporter response in cells derived from HOL Tg mice

**Figure 2:**
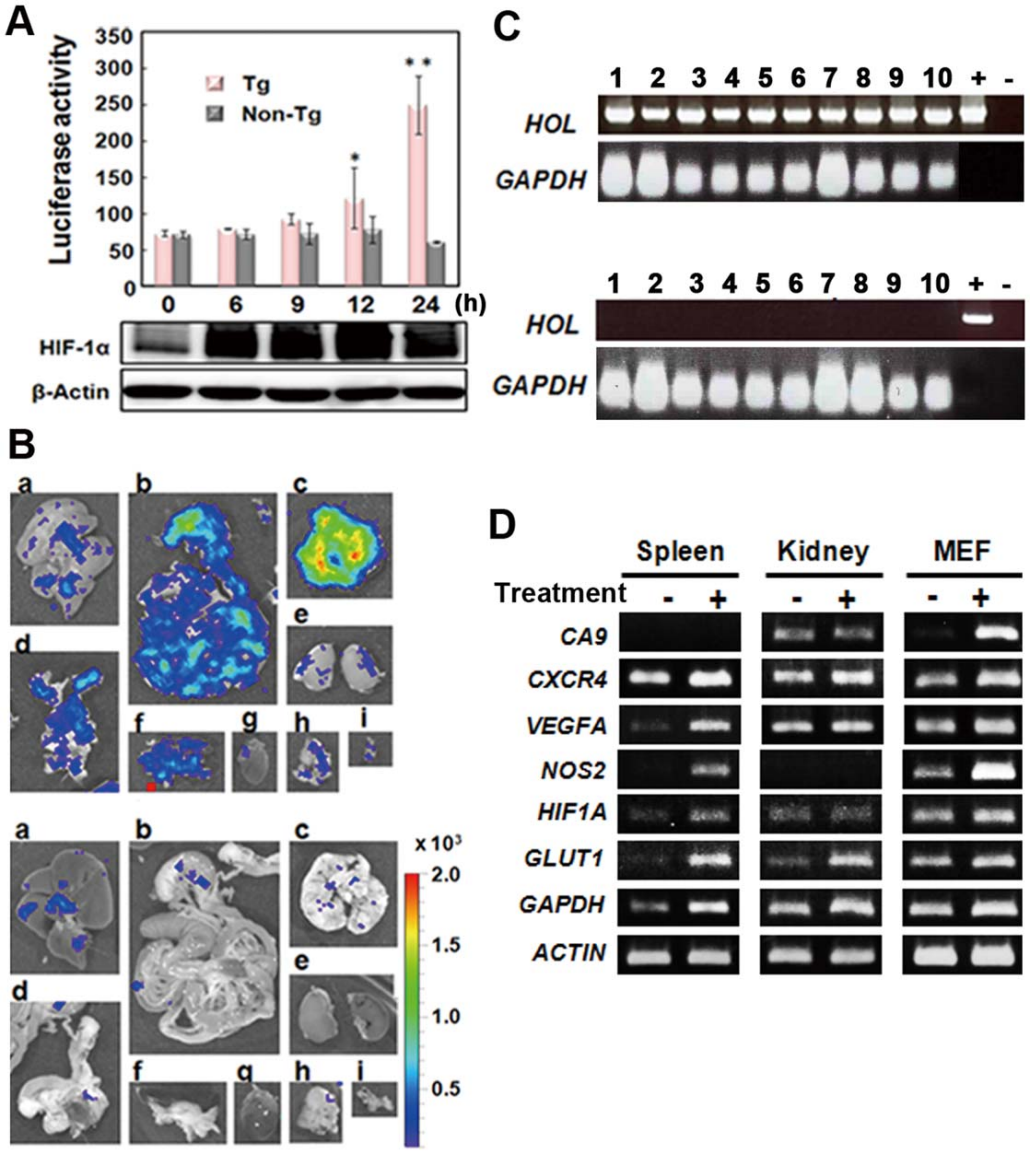
*In vitro* analysis of the *HOL* reporter response to HIF-1 activation. (A) MEF cells from FVB/HOL (Tg) and FVB/N (non-Tg) mice were cultured under hypoxic conditions (1% O_2_) for the indicated time, and luciferase activity was measured. The experiments were performed in triplicate, and the mean luciferase activity ± SD is shown in the graph. *P<0.05, **P<0.005. (B) FVB/HOL mice were peritoneally injected with PG, and 2 h later RNA was isolated from the indicated tissues. RT-PCR analysis was performed with the isolated RNA. Glyceraldehyde-3-phosphate dehydrogenase was used as an internal control. +, indicates a transgene used as a template; −, indicates no template. The experiment was performed in triplicates, and the representative data are shown. (C) FVB/HOL (Tg) and FVB/N (non-Tg) mice were peritoneally injected with PG, and 2 h later the mice were injected with luciferin through the tail vain; 2 min later, the tissues were isolated and observed by *ex vivo* bioluminescent imaging. a, liver; b, intestine; c, lung; d, uterus; e, kidney; f, spleen and pancreas; g, heart; h, thymus; i, bladder.

In an initial study, we examined the *in vitro* and *ex vivo* reporter response to hypoxia-induced HIF-1 activity by using cells isolated from FVB/HOL mice. Mouse embryo fibroblast (MEF) cells were cultured under hypoxic conditions, and their luciferase activity was measured. Luciferase activity in the MEF cells was expressed at significantly higher levels relative to that of the control 12 h after exposure to hypoxia (1% O_2_) (Figure 2A). RT-PCR was then used to examine the expression of the transgene in tissues isolated from FVB/HOL mice 2 h after peritoneal injection of PG. All examined tissues were found to express the reporter transcript (Figure 2B). The expression of the luciferase reporter was further confirmed by using tissues isolated from the FVB/HOL mice 2 h after the peritoneal injection of PG. Significant bioluminescence was detected in the tissues from the PG-treated FVB/HOL mice but not in those from PG-treated non-Tg mice (Figure 2C). These results demonstrated that the transgene in the HOL Tg mice could be used to monitor HIF-1 activity *in vitro* and *ex vivo*.

### Monitoring of HIF-1 activity *in vivo*

**Figure 3:**
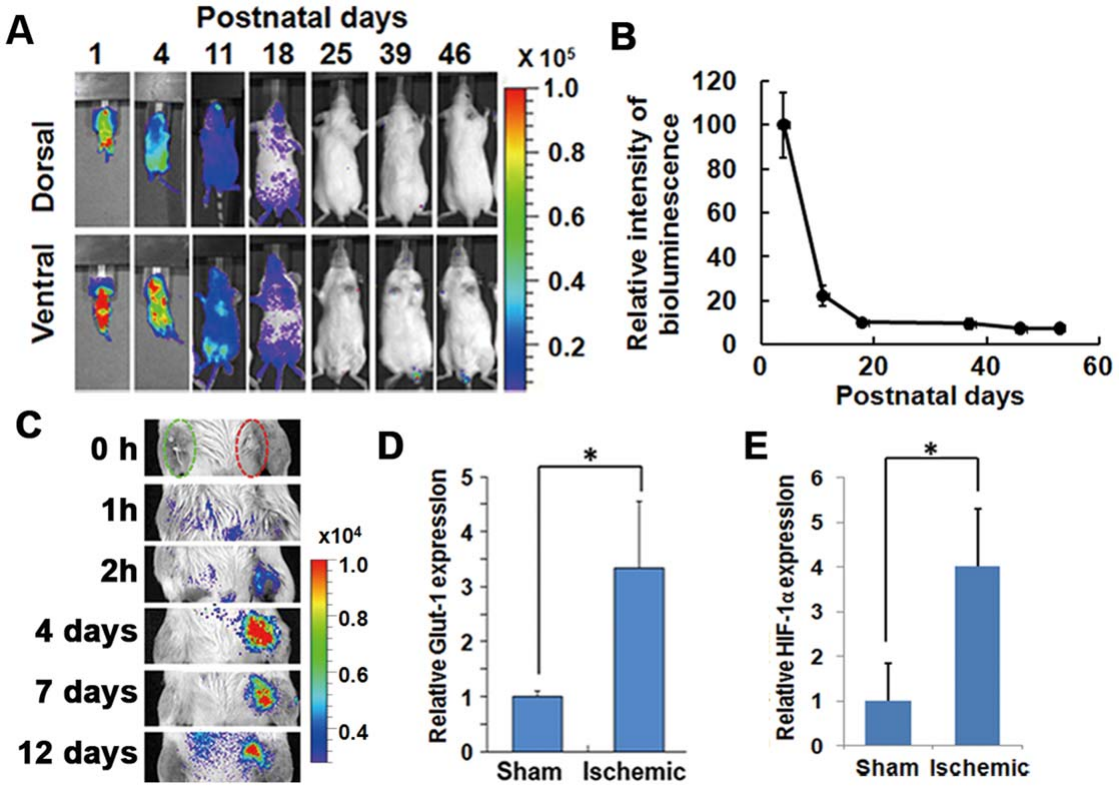
*In vivo* analysis of the *HOL* reporter response to HIF-1 activation. (A, B) The HIF-1 activity after birth. FVB/HOL mice were peritoneally injected with luciferin on the indicated day after birth, and bioluminescent images were acquired 20 min later. (A) Representative images are shown. (B) Total whole body photon counts were measured 4, 11, 18, 25, 39, 46, and 53 days after birth, and the photon counts (photon counts/s/mm^3^) in the region of interest are shown as values normalized to that of day 4 (*n* = 5). Results are given as the means ± SEM. (C) The femoral artery was ligated and cut without damaging the nevus femoralis. A sham operation was performed on the ipsilateral side. Changes in bioluminescence were monitored sequentially at indicated time periods after the operation. (D, E) Seven days after the ligation surgery of the femoral vessels, RNA was isolated from the skeletal muscle of the mice legs, and semi-quantitative real-time RT-PCR was performed. *GLUT1* (D) and *HIF-1α* (E), known as HIF-1 target genes, from the mRNA of the ischemic hindlimb muscle of the mice was quantified and normalized relative to the 18S rRNA level (*n* = 3). Results are given as the means ± SD. *P<0.05.

Next, we examined the reporter response to HIF-1 activity *in vivo*. Initially, we monitored bioluminescence after birth. The bioluminescence signal was the highest 1 day after birth and quickly decreased afterwards during the first week (Figures 3A and 3B), while no bioluminescence was detected in non-Tg mice (Figure S3). Within the third week after birth, the bioluminescence signal was at a basal level for the entire body (Figures 3A and 3B) and was at the same background level as that of non-Tg mice (Figure S1). Subsequently, we monitored bioluminescence after ischemic treatment of 8-week-old mice. The femoral artery was ligatured proximally and distally and then cut between the ligatures without damaging the femoral nerve. A sham operation was performed on the ipsilateral side. After the operation, bioluminescence imaging was performed sequentially, starting from 0 h till the next 12 days (Figure 3C). A significantly high bioluminescence signal was detected in the ligatured leg 2 h after the operation and continued until 12 days after the operation. For confirming the increase of HIF activity in the ligatured leg, the mRNA of ischemic hind limb tissues was isolated 7 days after the operation. The level of GLUT1, a HIF-1 target gene, was then quantified by using semi-quantitative real-time RT-PCR. GLUT1 expression was found to be significantly increased in the ischemic hind limb compared to that in the sham-operated one (Figure 3D). These results showed that HIF-1 activation can be monitored with HOL Tg mice.

### Incidence of MNU-induced tumors in Tg F1 mice with *H-ras* and *HOL* transgenes

To investigate HIF-1 activity during tumor development *in vivo*, we mated HOL mice with rasH2 mice to generate rasH2-HOL mice. Because the black coat of rasH2 mice is an obstacle for imaging whole body bioluminescence, we first generated B6-albino-rasH2 mice; these were established by mating rasH2 mice to B6-*Tyr^c^* (B6-albino) mice, which carry a tyrosinase gene mutation that results in a recessive albino phenotype. Before monitoring HIF activity in rasH2-HOL mice after MNU treatment, we examined the influence of *HOL* and *Tyr^c^* transgenes and the weekly treatment of luciferin on the incidence of MNU-induced tumors in rasH2 mice. In accordance with an established 6-month protocol for a carcinogenicity assessment of rasH2 mice, the white-coated B6-albino-rasH2 mice were mated with the BALB/HOL mice, and the MNU-induced tumor incidence was examined in the F1 mice, which segregated according to the expected 4 genotypes (Ras^−^HOL^−^, Ras^−^HOL^+^, Ras^+^HOL^−^, and Ras^+^HOL^+^) (Figure S4). The MNU-induced tumors that were observed in the mice of each group are shown in Table 1. A high incidence of papilloma and carcinoma were observed in the Ras^+^HOL^−^ and Ras^+^HOL^+^ mice but not in the Ras^−^HOL^−^ and Ras^−^HOL^+^ mice. The tumor incidence in the Ras^+^HOL^−^ and Ras^+^HOL^+^ mice was the same as that observed in the rasH2 mice [23], [24]. These mice were intraperitoneally administrated luciferin every week. These results indicate that the *HOL* and *Tyr^c^* transgenes and the luciferin treatment do not influence MNU-induced tumor incidence in rasH2 mice.

**Table 1:** Tumor incidence rate in transgenic mice after *N*-methyl-*N*-nitrosourea treatment.

Genotype	Squamous cell carcinoma/papilloma (Stomach)	Squamous cell papilloma (Skin)	Malignant lymphoma (Hematopoietic system)
Ras^−^HOL^−^	6.7%*	0%	60%
Ras^+^HOL^−^	87.5%	37.5%	50%
Ras^−^HOL^+^	0%	0%	55.6%
Ras^+^HOL^+^	62.5%	37.5%	75%

*Tumor incidence rate. Carcinogenicity test of 15 mice from each of the following offspring type: CB6F1-albino (Ras^−^HOL^−^), CB6F1-albino-rasH2 (Ras^+^HOL^−^), C6F1-albino-HOL (Ras^−^HOL^+^), and CB6F1-albino-rasH2-HOL (Ras^+^HOL^+^) were performed by administering *N*-methyl-*N*-nitrosourea. The mice that were alive at the end of the experimental period were necropsied, and systemic visual observations were performed.

### Bioluminescence imaging of HIF-1 activity in Tg mice with both *Ras* and *HOL* transgenes after MNU treatment

**Figure 4:**
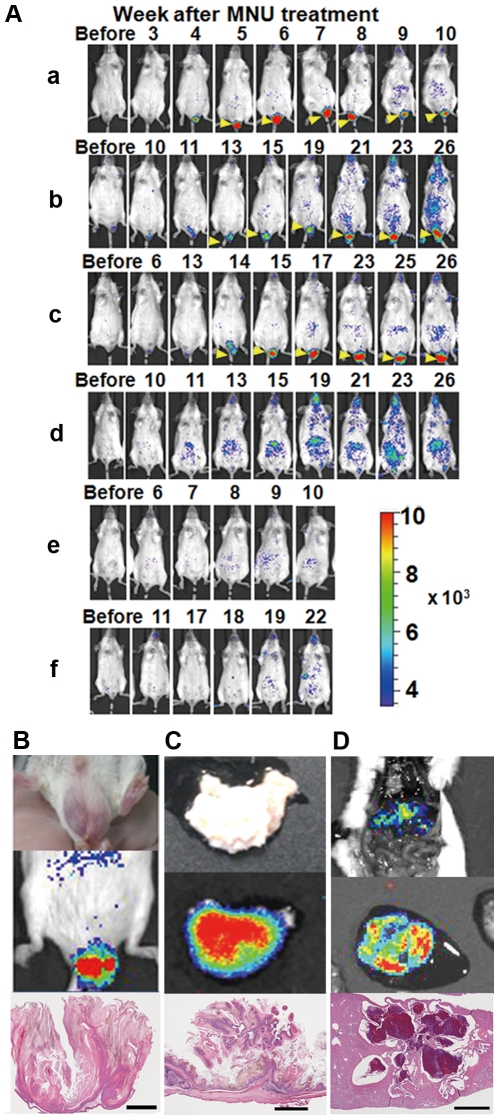
*In vivo* bioimaging of cancer formation. (A) Double (Ras^+^HOL^+^) transgenic mice were peritoneally injected with MNU and bioluminescence images were acquired every week. Bioluminescent signals from the papilloma of the scrotum are indicated by yellow arrowheads. (B) Brightfield image (upper left), *in vivo* bioluminescence image (upper right), and tumor section (bottom) of the squamous cell papilloma of the scrotum from mouse c. (C) Brightfield image (upper left), *ex vivo* bioluminescence image (upper right), and tumor section (bottom) of the squamous cell papilloma/carcinoma of the stomach from mouse b. (D) *Ex vivo* bioluminescence image (upper) and the tumor section (bottom) of the hemangioma of the liver from mouse c. bar = 1 mm.

Finally, we monitored HIF activity in MNU-treated Ras^−^HOL^+^ and Ras^+^HOL^+^ Tg mice by *in vivo* bioluminescence imaging. Since FVB/HOL mice repeatedly showed stronger bioluminescence signals than the BALB/HOL ones (Figure S5), we used mice from the FVB background in the assay. Six mice (8–9 weeks old, 3 males and 3 females) were intraperitoneally injected with MNU and then imaged every week until the 26^th^ week. All the Ras^+^HOL^+^ mice (a–f) showed a significant bioluminescence signal (Figure 4A), while none of the Ras^−^HOL^+^ mice had a significant bioluminescence signal (Figure S6). Three Ras^+^HOL^+^ mice (a, e, and f in Figure 4A) and 2 Ras^−^HOL^+^ mice (g and h in Figure S6) died during the observation period. Either at the time of death or at the end of the experimental period, all the mice were examined for tissue lesions, with the major tissues investigated by histopathological analysis. The results are summarized in Table 2. A strong bioluminescence signal was detected in papillomas of the scrotum as early as the fifth week in all of the male Ras^+^HOL^+^ mice (yellow arrowheads in Figures 4A, 4B, and S7). Moreover, all the Ras^+^HOL^+^ mice (a–f) had significant bioluminescence signals in the abdominal region as early as the ninth week (Figure 4A). *Ex vivo* imaging was also performed for the mice still alive at the end of the experimental period in order to confirm that the tissues had a bioluminescence signal (Figures 4C and 4D). The tissues with the bioluminescence signal were histopathologically analyzed and diagnosed as squamous cell papilloma/carcinoma (Figure 4C) or adenocarcinoma in the forestomach and hemangioma in the liver (Figure 4D). These results strongly suggest that HIF-1 activity is closely associated with cancer formation.

**Table 2:** Histopathological analysis of rasH2-HOL and FVB/HOL mice treated with *N*-methyl-*N*-nitrosourea.

	Mouse	Sex	Tissues	Histopathological diagnosis
RasH2-	A	♂	Stomach	Squamous cell papilloma
HOL			Scrotum	Squamous cell papilloma
(Ras^+^HOL^+^)	B	♂	Stomach	Squamous cell papilloma
			Legs	Squamous cell papilloma
			Scrotum	Squamous cell papilloma
	C	♂	Lung	Alveolar/bronchiolar adenoma
			Liver	Hemangioma
			Stomach	Squamous cell papilloma/adenocarcinoma
			Scrotum	Squamous cell papilloma
	D	♀	Stomach	Squamous cell papilloma/carcinoma
	E	♀	Hematopoietic system	Malignant lymphoma
			Stomach	Squamous cell papilloma
	F	♀	Hematopoietic system	Malignant lymphoma
			Stomach	Squamous cell papilloma
FVB/HOL	G	♂	Hematopoietic system	Malignant lymphoma
(Ras^−^HOL^+^)	H	♀	Lung	Alveolar/bronchiolar adenoma
	I	♂		No significant lesion
	J	♂		No significant lesion
	K	♀		No significant lesion
	L	♀		No significant lesion

## Discussion

This is the first report of a mouse model for *in vivo* bioimaging of endogenous cancer formation, and it was accomplished by using 2 Tg mouse lines, HOL and rasH2.

The HOL Tg mice sensitively provided information on HIF-1 transcriptional activity at the site of disease as well as in healthy tissues (Figure 3). During the first week after birth, HIF-1 activity was high, consistent with previous reports that HIFs and specific HIF target genes are actively involved in maturational processes during embryonic, fetal, and postnatal development by modulating cell differentiation, vascular development, angiogenesis, and metabolic homeostasis [27], [28]. No significant signal was detected in the body >3 weeks after birth (Figures 3A and 3B), indicating that continuous HIF-1 activity is not necessary for normal adult tissues and that high levels of sustained HIF-1 activity would indicate an abnormal status for tissues. Although there are significant number of reports that HIF-1 activity plays key role in tumor growth and malignancy, little information is available on the role of HIF-1 transcriptional activity during endogenous tumor development. By using double (Ras^+^HOL^+^) Tg mice, we successfully monitored MNU-induced carcinogenesis (Figure 4). The double (Ras^+^HOL^+^) Tg mice developed papillomas and carcinomas at a frequency similar to what had been observed for rasH2 mice treated with MNU (Table 1, Table 2 and Ref. [23], [24]). The results demonstrate that HIF-1 activity was detected in most, if not all, cancers and papillomas as early as 9 weeks after MNU treatment (Figure 4A), indicating that HIF-1 activity is involved in papilloma formation and in the early stages of tumor development. These results strongly suggest that the Ras^+^HOL^+^ Tg mice would be useful for studying the initial events of tumor formation *in vivo*.

Mice c and h had alveolar/bronchiolar adenoma in the lung, while no detectable bioluminescence signal was observed around the chest. One explanation is that the small size of the cancers resulted in a signal that was below the detection limits of the existing imaging instruments (Figure S8). Bioluminescence signals from deep organs are difficult to detect; the externally detected signal from the lung (∼5 mm depth) has been shown to be about 1/80 of that from the skin (∼0.5 mm depth) [29]. Moreover, when we performed immunohistochemical analysis, we failed to detect luciferase protein expression in the tissues that showed significant bioluminescent signal during the *in vivo* and *ex vivo* observations. This is probably due to the short half-life (17 min) of the ODD-luciferase reporter protein; the ODD polypeptide fused to the luciferase accelerates the degradation of the luciferase, allowing the detection of real-time HIF-1 activity *in vivo* [18].

Recently, a Tg *ROSA26* ODD-Luc/+ mouse line for the *in vivo* monitoring of HIF PHD activity was reported [30]. In these mice, the reporter protein ODD (530–652)-luciferase, under the control of a cytomegalovirus promoter, was constitutively expressed at a high level in all tissues, with PHD function monitored through the reduction in luciferase activity [30]. The effects of regulatory factors other than PHDs on HIF-1 are also important in the monitoring of HIF-1 activity in cancerous cells [5]–[17]. Therefore, it would be difficult to precisely detect tumor formation by the Tg mice.

Our findings indicate that Ras^+^HOL^+^ mice hold significant potential as a novel, alternative *in vivo* model to the lifetime mouse bioassay for determining the carcinogenic potential of chemicals. The Tg rasH2 mouse model has been one of the most thoroughly tested *in vivo* alternatives to the lifetime mouse bioassay for both nongenotoxic and genotoxic compounds [31], [32]. As regulatory and industry experience with the use of this model in carcinogenicity testing has grown, there has been increasing scientific and regulatory consensus that the model has added value to drug safety testing and risk assessment for the carcinogenicity of pharmaceuticals [31], [32]. In particular, it has been used in the internal decision-making process in terms of whether to advance a compound or to investigate the potential mechanisms of carcinogenicity [33]. Bioassays using Ras^+^HOL^+^ mice may be able to further shorten the assessment period. If so, it would contribute both to the earlier termination of clinical programs for nonviable compounds and to the earlier redirection of a development program for a viable candidate, leading to the acceleration of new drug development. More importantly, from the point of view of animal welfare, the early detection of cancer would help avoid unnecessary pain and stress for the mice, and the smaller number of required animals would contribute to the goals of reducing and refining animal use. Although further studies using other carcinogens are needed for comparing the sensitivity between rasH2 and Ras^+^HOL^+^ mice, the results presented here demonstrate that HIF-based *in vivo* bioluminescence imaging is a reliable strategy for detecting carcinogenesis.

Collectively, the results suggest that the Tg mice presented here would be useful as a mouse model for *in vivo* bioimaging to investigate the onset and progression of cancers as well as other diseases related to HIF-1 activity and for developing drugs and treatment strategies for those diseases.

## Materials and Methods

### Ethics statement

All animal experiments were performed with the approval of the Animal Ethics Committees of the Central Institute for Experimental Animals (CIEA; No. 08028), Kyoto University (No. Mde Kyo 09247), and Tokyo Institute of Technology (No. 2010008) and in accordance with the Ethical Guidelines for Animal Experimentation of the CIEA, Kyoto University, and Tokyo Institute of Technology.

### Development of HOL transgenic (Tg) mouse

All mice used to generate the present Tg mice were purchased from CLEA Japan Inc. (Tokyo, Japan). Construction of the transgene, *5HRE-CMVmp-NLS-ODD(548–603)-(firefly luciferase)*, which contains 5 tandem repeats of the hypoxia-response element, a minimal cytomegalovirus promoter, and cDNAs encoding the nuclear localization signal sequence of the Simian vacuolating virus 40 T-antigen, the human hypoxia-inducible factor 1α (HIF-1α) oxygen-dependent degradation domain 548–603 (ODD_548–603_), and firefly luciferase (Figure 1A), has been reported elsewhere [18]. The Tg mice were developed by microinjecting the 2.2 kb transgene fragment into fertilized eggs with a C57BL/6J mice background. Two lines of Tg mice were obtained. To facilitate bioimaging, the coat color was exchanged by further backcross mating of BALB/cA mice for more than 17 generations. The Tg mouse line used in the further experiments was named BALB/HOL (formally, C.C6-Tg[HRE-HIF1AODD/luc]2Skk/JKyo). Furthermore, the same transgene was injected into the fertilized eggs with a FVB/N mouse background. A Tg mouse line was established by mating these syngeneic mice. This line (formally, FVB-Tg[HRE-HIF1AODD/luc]2Skk/NKyo) is described in the text as FVB/HOL.

### Development of the rasH2-albino and rasH2-albino/HOL Tg mouse

C57BL/6J-Tg(HRAS)2/Jic (rasH2), C57BL/6J-*Tyr^c^* (B6-albino), BALB/cAJcl, and MCH:ICR/Jcl mice were used to develop the rasH2-albino and rasH2-albino/HOL Tg mice. The rasH2 mice were maintained at CIEA. The B6-albino mice were purchased from Charles River Inc. (Kanagawa, Japan). BALB/cAJcl and MCH:ICR/Jcl mice were purchased from CLEA Japan, Inc. (Tokyo, Japan). All mice were reared in plastic cages (190×245×130 mm; CLEA Japan), with 3 animals per cage, in an animal room with controlled temperature at 24°C±2°C, humidity at 60%±15%, 12–25 air ventilations/h, and 12 h of artificial lighting (08:00–20:00). Commercial diet was supplied *ad libitum*. Filtered tap water was sterilized with ultraviolet (UV) radiation and supplied *ad libitum* for drinking using a water bottle. The mice were subjected to a 1 week acclimatization period before treatment.

To generate rasH2 mice with white fur to facilitate bioimaging, B6-albino mice were mated to the rasH2 mice. Offspring carrying the *rasH2* transgene were selected by genotyping with a multiplexed PCR to amplify the 510- and 130-base pair (bp) Tg products and the 260-bp non-Tg product as previously described [23]. For genotyping, genomic DNA was extracted from the tails of the resulting offspring using an automatic DNA extraction apparatus (MagExtractor System MFX-9600, Toyobo, Osaka, Japan) according to the manufacturer's instructions. The rasH2-albino mice were obtained by further intercross mating of selected offspring carrying the *ras*H2 transgene and by checking their genotypes and fur color.

The rasH2-albino/HOL Tg mice were generated by *in vitro* fertilization between sperm from rasH2-albino male mice and unfertilized eggs from BALB/HOL Tg mice. The fertilized eggs were transplanted into the oviducts of pseudopregnant MCH:ICR female mice to obtain offspring. Mice carrying the *HOL* and *rasH2* transgenes were genotyped using the respective PCR primer sets to amplify the transgenes. For genotyping of the ODD transgene, a forward primer (5′-AGACTTGGAGATGTTAGCTCCCTATATCC-3′) and a reverse primer (5′-TCTTGTCCCTATCGAAGGACTCTGGCACA-3′) were used, and the resulting specific product size was 700 bp.

### Carcinogenicity test by *N*-methyl-*N*-nitrosourea administration

Fifteen mice each of the offspring, CB6F1-albino (Ras^−^HOL^−^), CB6F1-albino-rasH2 (Ras^+^HOL^−^), CB6F1-albino-HOL(Ras^−^HOL^+^), and CB6F1-albino-rasH2-HOL (Ras^+^HOL^+^), were used for the carcinogenicity test by administration of *N*-methyl-*N*-nitrosourea (MNU) as previously described [24]. At 6–7 weeks of age, the mice were intraperitoneally injected once with 75 mg/kg of MNU (Nacalai Tesque, Kyoto, Japan) in citrate buffer at pH 4.5.

After administration, the mice were kept under daily observation to check for death. Mice were intraperitoneally injected with 10 mL/kg of d-luciferin (Promega, Madison, WI, USA) solution in phosphate-buffered saline (PBS]) every week. Twenty-six weeks after administration or at death or during an emergency, animals were necropsied and systemic visual observations were performed. Stomach (forestomach and glandular stomach), liver, kidneys, spleen, heart, lungs, thymus, and lymph nodes and any sites showing abnormalities were removed and fixed in 10% neutral buffered formalin. The tissues were embedded in paraffin and their 5-mm thick sections were prepared and stained with hematoxylin and eosin.

### *In vivo* and *ex vivo* bioluminescence imaging

*In vivo* and *ex vivo* bioluminescence imaging was conducted as described previously [34]. Briefly, for the *in vivo* photon counts assessing bioluminescence, mice were intraperitoneally injected with 10 mL/kg of d-luciferin solution (10 mg/mL in PBS) and placed in an IVIS Spectrum *in vivo* photon-counting device (Caliper Life Sciences, Alameda, CA, USA). Bioluminescence images were acquired 20 min after the intraperitoneal injection of d-luciferin. For *ex vivo* imaging, mice were sacrificed immediately after the *in vivo* imaging, and bioluminescence images of the body and organs were acquired within 10 min of the *in vivo* imaging. The following conditions were used for image acquisition: exposure time, 2 min (for *in vivo*) or 1 min (for *ex vivo*); lamp level, high; binning, medium: 8; field of view, 19×19 cm (for *in vivo*) and 12.9×12.9 cm (for *ex vivo*); f/stop, 1. The bioluminescence was analyzed using Living Image 3.10 software (Caliper Life Sciences). Because of the background bioluminescence of the untreated mice (from the d-luciferin injection) was <3,500 photon counts/s, we considered signals of >3,500 photon counts/s to be significant. The minimum and maximum bioluminescent signals (photons/s/cm^2^/sr) are 3,500 and 10,000, respectively, if not otherwise indicated in a figure with a rainbow bar scale.

### PG treatment

PG (3,4,5-trihydroxybenzoic acid propyl ester; Sigma-Aldrich Co., St. Louis, MO, USA) dissolved in ethanol (100 mg/mL) was diluted 1∶10 in PBS immediately before intraperitoneal administration (100 mg/kg).

### RT-PCR for reporter gene expression

The mice were dissected 2 h after PG administration. Tissues were removed, and total RNA samples were extracted from the tissues with TRIzol reagent (Invitrogen, Carlsbad, CA, USA) according to the manufacturer's protocol. Tissue cDNA was produced by reverse transcription, and the *HOL* reporter cDNA fragment was amplified by PCR using primer pairs 5′-ODD(+):AGA CTT GGA GAT GTT AGC TCC CTA TAT CC-3′ and 5′-Luc(−):TCT TGT CCC TAT CGA AGG ACT CTG GCA CA-3′.

### Southern blot analysis

Genomic DNA was prepared from the mouse tail by the conventional phenol/chloroform method. DNA samples were digested with *Pst*I applied to agarose gel electrophoresis and transferred to a nitrocellulose membrane. The transgene integrated into the mouse genome was detected with a DNA probe labeled with [^32^P]-dCTP by Ready Prime Labelling Kit (GE Healthcare, Waukesha, WI, USA) according to the manufacturer's protocol using the 2.2-kb transgene fragment.

### Luciferase reporter assay

Luciferase reporter assay used basically the same method as previously described [35]. Briefly, 10^5^ cells were first seeded onto 12-well plate in 2 mL of 5% fetal bovine serum/Dulbecco's Modified Eagle Medium and cultured overnight, then further cultured under hypoxic (1% O_2_) conditions for the indicated time. The cells were then washed with PBS and lysed with 100 µL passive lysis buffer (Promega) under hypoxic conditions. Luciferase activity was measured using a luminometer (Lumat LB 9507; Berthold Technologies, Bad Wildbad, Germany) after the addition of 50 µL of a substrate reagent (luciferin; Promega) to the 25 µL of cell lysate.

### Ischemic treatment

Mice were anesthetized by intraperitoneal pentobarbital injection (50 mg/kg), and the femoral vessels in right inguinal area were exposed. The femoral artery was ligated proximally and distally using 6-0 silk sutures and cut between the ligatures without damaging the nevus femoralis. The overlying skin was closed using 4-0 silk sutures. A sham operation was performed on the ipsilateral side.

### Measurement of *GLUT1* expression

Seven days after the ligation surgery of the femoral vessels, the mice (*n* = 3) were killed, and the RNA from the skeletal muscle of the mice legs was treated with DNase and purified using an RNeasy kit (QIAGEN, Venlo, The Netherlands). First-strand synthesis and real-time PCR reaction were performed using QuantiTect SYBR Green PCR Kit (Qiagen) following the protocol provided by the company. The PCR reaction and detection were performed using a 7300 real-time PCR system (Applied Biosystems, Foster City, CA, USA). The PCR primers were purchased from QIAGEN. Using semi-quantitative real-time RT-PCR, *GLUT1*, known as the HIF-1 target gene, mRNA of the mice ischemic hindlimb muscle was quantified. The RT-PCR data were quantified and normalized relative to the 18S rRNA level. The fold induction was calculated on the average value of the ipsilateral non-ischemic legs. Results shown represent mean ± standard deviations.

### Statistical analysis

Statistical analyses used the Student *t* test. Values of *P*<0.05 were considered statistically significant. Pearson correlation coefficient was used to evaluate the correlation of the data sets.

## Supporting Information

Figure S1 **Background bioluminescence of FVB/N mice.** Wild-type FVB/N mice were either injected peritoneally with PG or were not injected with PG (none). Two hours later luciferin was injected and bioluminescence images were acquired 20 min after luciferin injection. The image acquisition conditions were the same as those described for Figure 1b.
(TIF)

Figure S2 **Fluorescence in situ hybridization analysis of the FBV/HOL genome.** G-band (left) and R-band (right) analyses of the chromosomes. The arrowhead shows the signal on the R-band. The map of chromosome 19 is shown below. Experimental method was shown in Text S1.
(TIF)

Figure S3 **Bioluminescence in newborns.** One-day-old FVB/HOL (Tg) and FVB/N (Non-Tg) mice were injected with d-luciferin (0.5 mg/50 µL/body). After 10 min, the bioluminescence images were acquired.
(TIF)

Figure S4 **Generation of mice used in the 6-mo protocol for carcinogenicity assessment.** A total of 300 offspring were produced by *in vitro* fertilization of sperm from B6-*Tyr* ^c^-rasH2 male mice into unfertilized eggs from BALB/HOL female mice, followed by transplantation into the oviducts of pseudo-pregnant MCH:ICR female mice. Results of the genotyping of 125 females are shown in Table 1. The production rates of mice with Ras^+^HOL^+^ and Ras^−^HOL^+^ were 19.2% and 17.8%, respectively, that were slightly lower than the ratio expected according to Mendelian law.
(TIF)

Figure S5 **Comparison of the strength of the bioluminescent signal between BALB/HOL and FVB/HOL mice (representative images).** Upper panels show photos of the papillomas from the rasH2-BALB/HOL and the rasH2-FVB/HOL mice. Lower panels show bioluminescence images of the papillomas. The papillomas in the rasH2-BALB/HOL mice were much larger than the ones in the rasH2-FVB/HOL mice, whereas the bioluminescent signal in the papillomas of the rasH2-FVB/HOL mice was always stronger.
(TIF)

Figure S6 **Images of the FVB/HOL mice used in the 6-mo protocol for carcinogenicity assessment.** (G–L) The FVB/HOL mice did not show significant bioluminescent signals during the period of the experiment. The image acquisition conditions were the same as those for Figure 4A. Mice G and H died 22 weeks after the *N*-methyl-*N*-nitrosourea (MNU) treatment.
(TIF)

Figure S7 **Images of papillomas.** Photos of the papillomas in mice A and B were taken 18 weeks after the *N*-methyl-*N*-nitrosourea treatment.
(TIF)

Figure S8 **The alveolar/bronchiolar adenoma from mouse C.** Bright field image of the lung (left) and tumor section (right, ×50) of the alveolar/bronchiolar adenoma (yellow arrow head in the left panel) from mouse C. Bar = 3 mm.
(TIF)

Text S1 **FISH analysis.**
(DOCX)
