# Supplementary figures and images for: Detection of the Onset of Ischemia and Carcinogenesis by Hypoxia-Inducible Transcription Factor-Based In Vivo Bioluminescence Imaging
Source: PLoS One. 2011 Nov 10;6(11):e26640. doi: 10.1371/journal.pone.0026640 (PMC3213102; doi:10.1371/journal.pone.0026640)

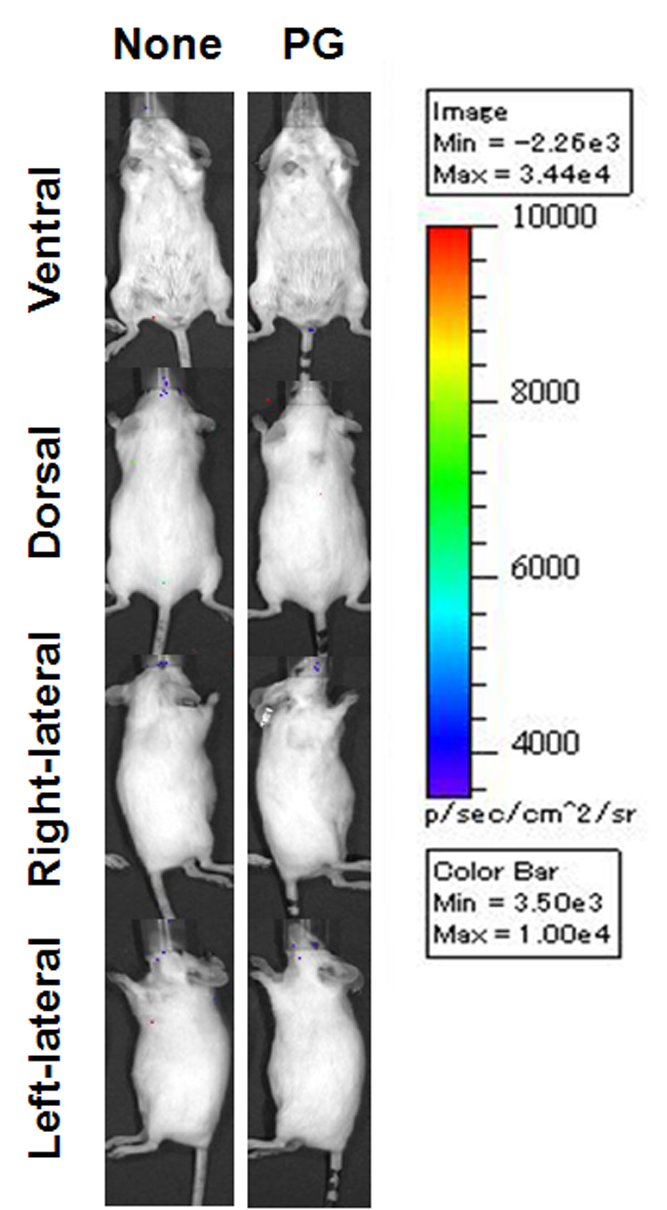

Supplement: Figure S1 — Background bioluminescence of FVB/N mice. Wild-type FVB/N mice were either injected peritoneally with PG or were not injected with PG (none). Two hours later luciferin was injected and bioluminescence images were acquired 20 min after luciferin injection. The image acquisition conditions were the same as those described for Figure 1b. (TIF) [file pone.0026640.s001.tif]

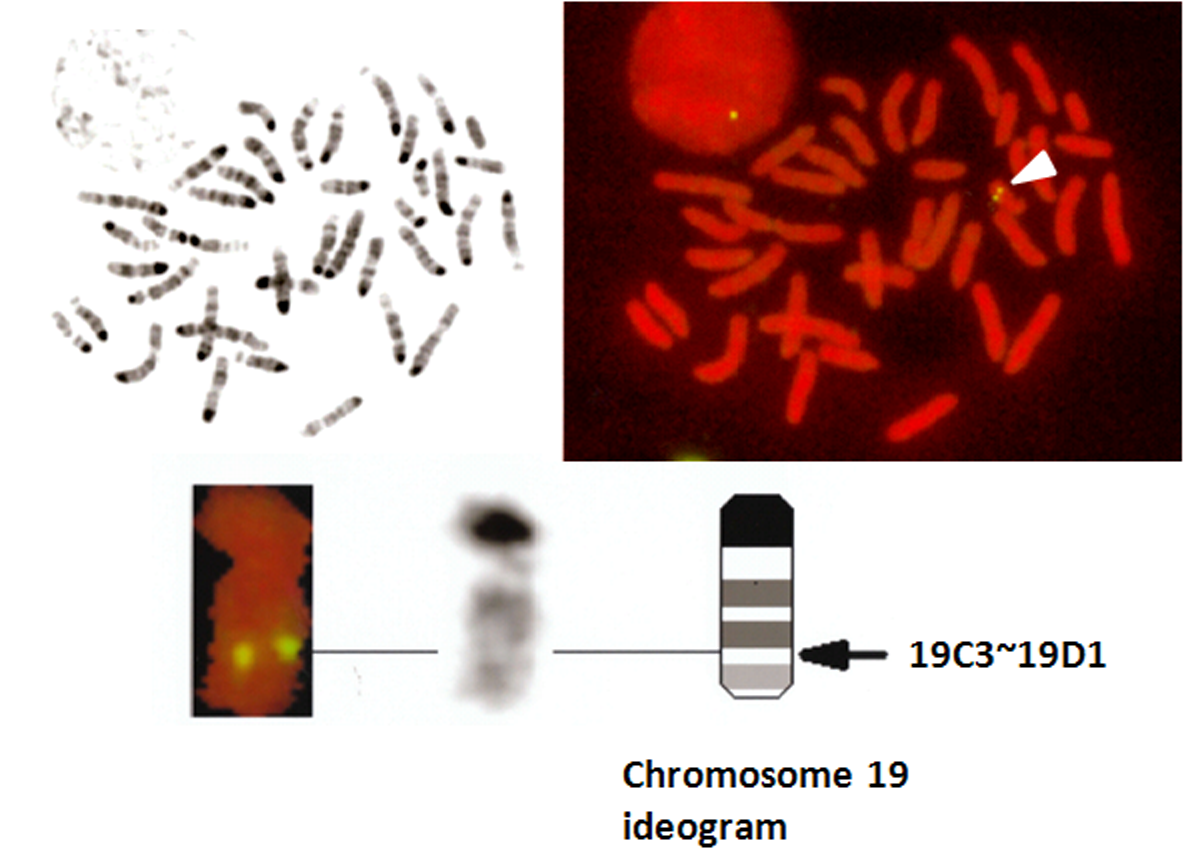

Supplement: Figure S2 — Fluorescence in situ hybridization analysis of the FBV/HOL genome. G-band (left) and R-band (right) analyses of the chromosomes. The arrowhead shows the signal on the R-band. The map of chromosome 19 is shown below. Experimental method was shown in Text S1. (TIF) [file pone.0026640.s002.tif]

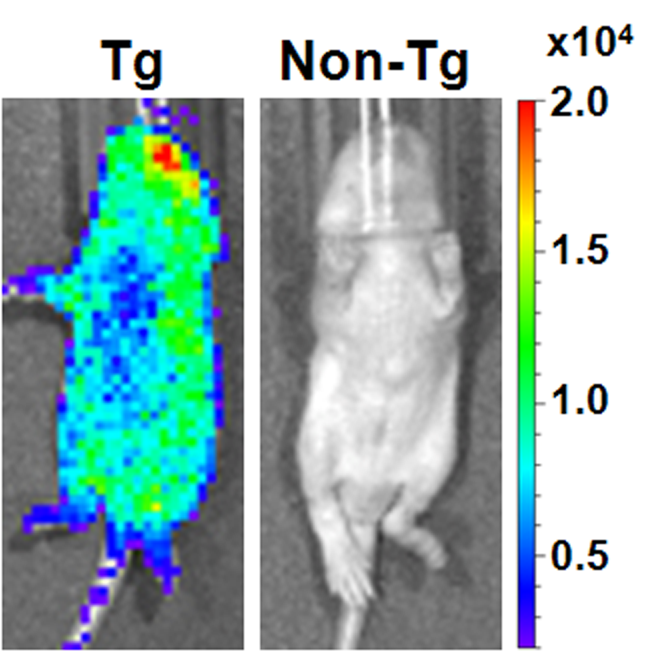

Supplement: Figure S3 — Bioluminescence in newborns. One-day-old FVB/HOL (Tg) and FVB/N (Non-Tg) mice were injected with d-luciferin (0.5 mg/50 µL/body). After 10 min, the bioluminescence images were acquired. (TIF) [file pone.0026640.s003.tif]

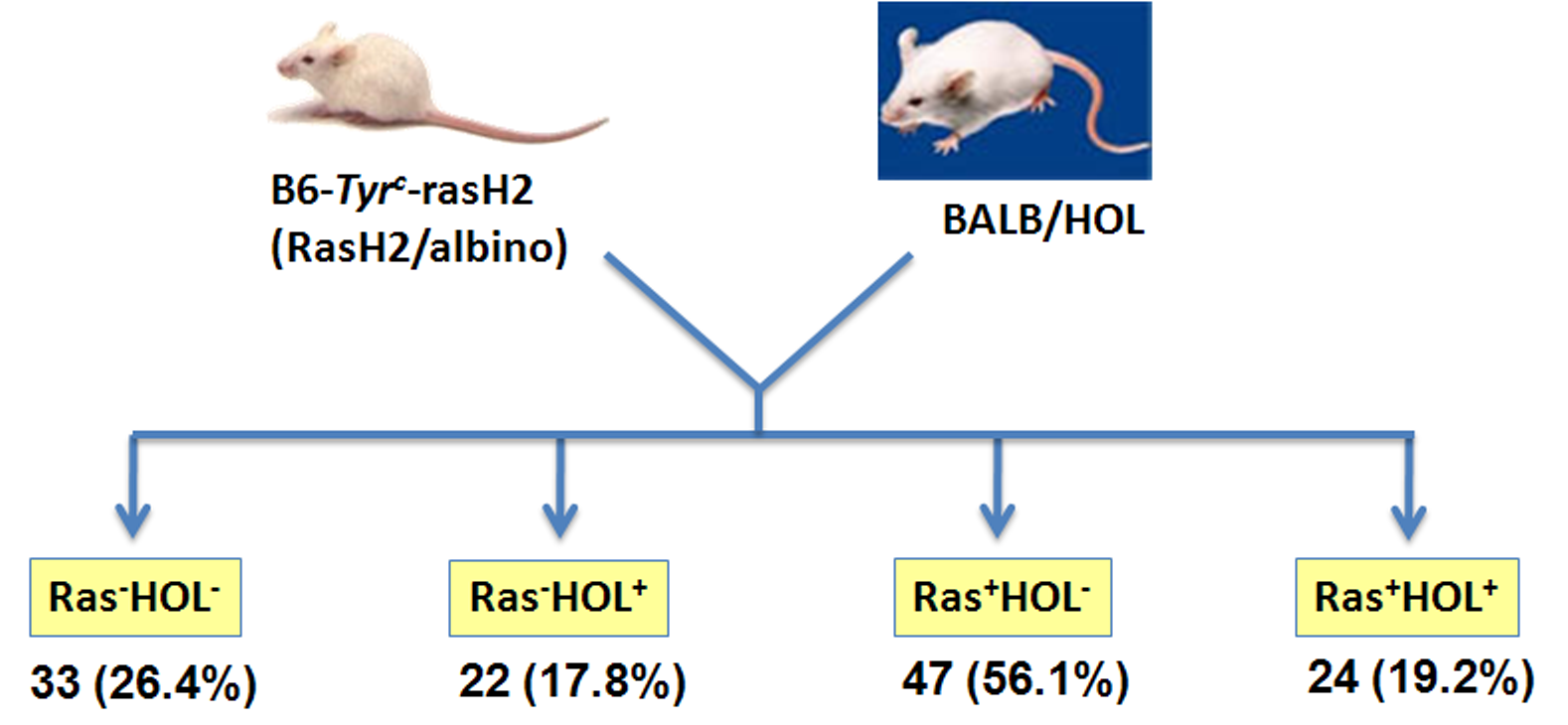

Supplement: Figure S4 — Generation of mice used in the 6-mo protocol for carcinogenicity assessment. A total of 300 offspring were produced by in vitro fertilization of sperm from B6-Tyr c-rasH2 male mice into unfertilized eggs from BALB/HOL female mice, followed by transplantation into the oviducts of pseudo-pregnant MCH:ICR female mice. Results of the genotyping of 125 females are shown in Table 1. The production rates of mice with Ras+HOL+ and Ras−HOL+ were 19.2% and 17.8%, respectively, that were slightly lower than the ratio expected according to Mendelian law. (TIF) [file pone.0026640.s004.tif]

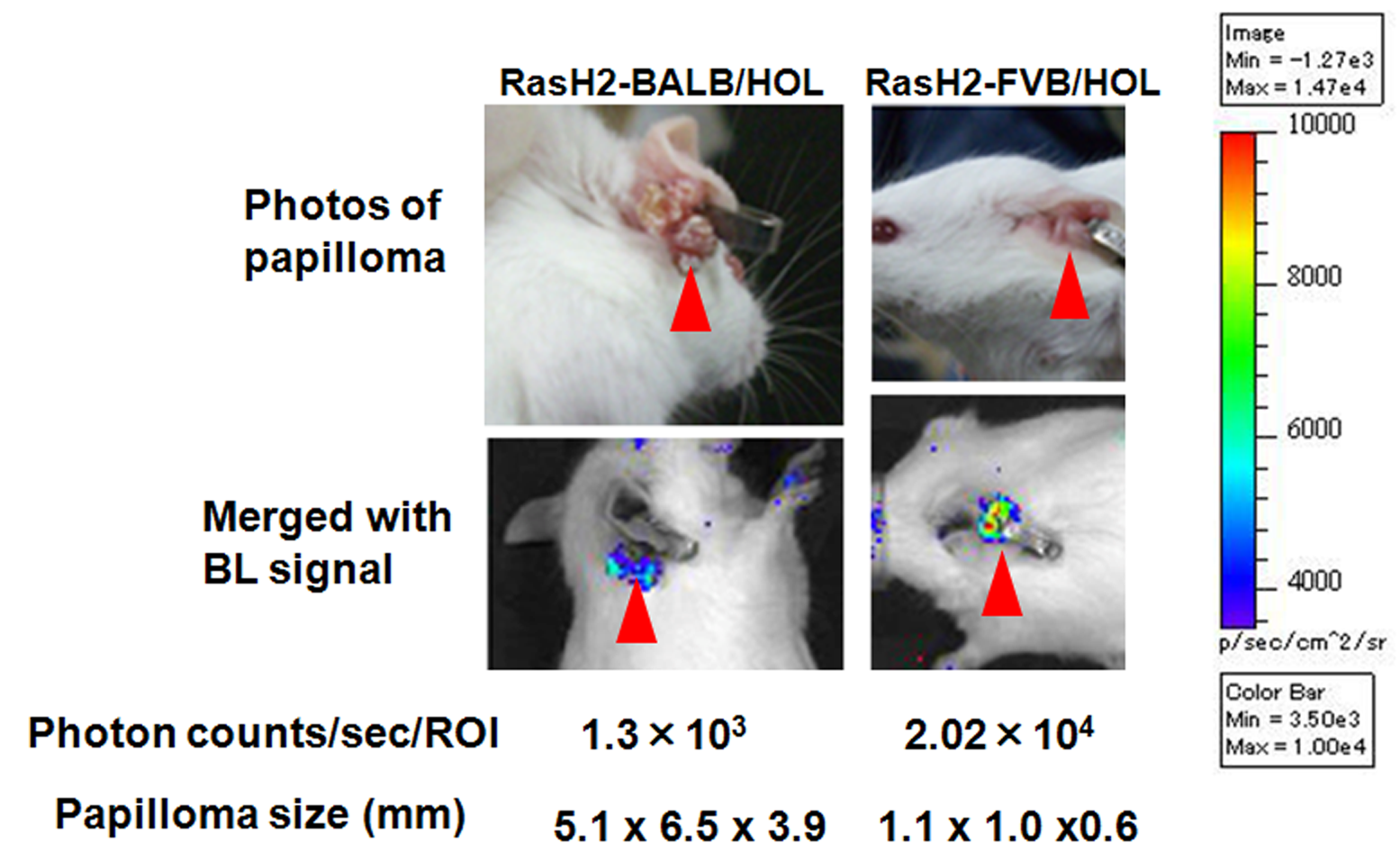

Supplement: Figure S5 — Comparison of the strength of the bioluminescent signal between BALB/HOL and FVB/HOL mice (representative images). Upper panels show photos of the papillomas from the rasH2-BALB/HOL and the rasH2-FVB/HOL mice. Lower panels show bioluminescence images of the papillomas. The papillomas in the rasH2-BALB/HOL mice were much larger than the ones in the rasH2-FVB/HOL mice, whereas the bioluminescent signal in the papillomas of the rasH2-FVB/HOL mice was always stronger. (TIF) [file pone.0026640.s005.tif]

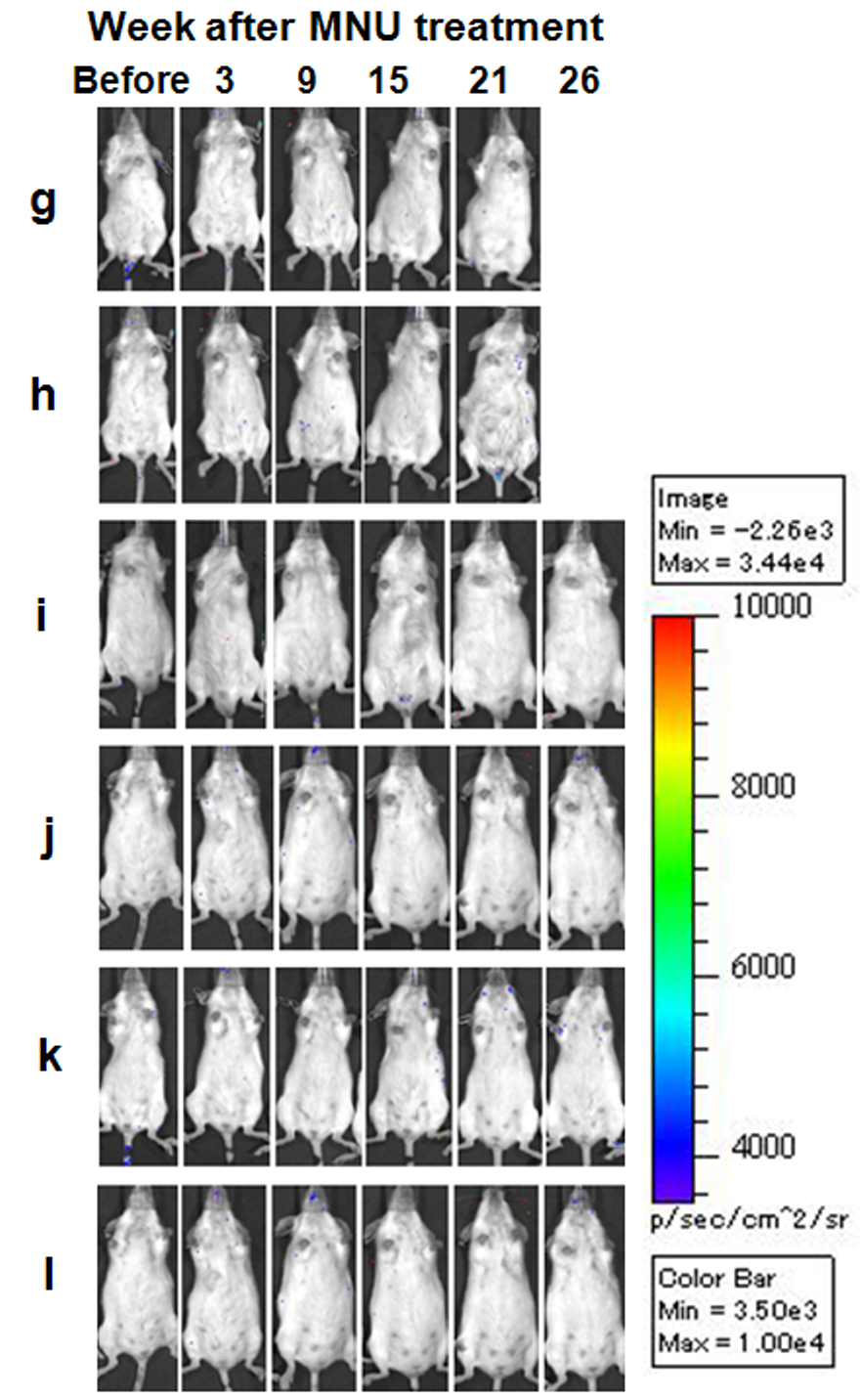

Supplement: Figure S6 — Images of the FVB/HOL mice used in the 6-mo protocol for carcinogenicity assessment. (G–L) The FVB/HOL mice did not show significant bioluminescent signals during the period of the experiment. The image acquisition conditions were the same as those for Figure 4A. Mice G and H died 22 weeks after the N-methyl-N-nitrosourea (MNU) treatment. (TIF) [file pone.0026640.s006.tif]

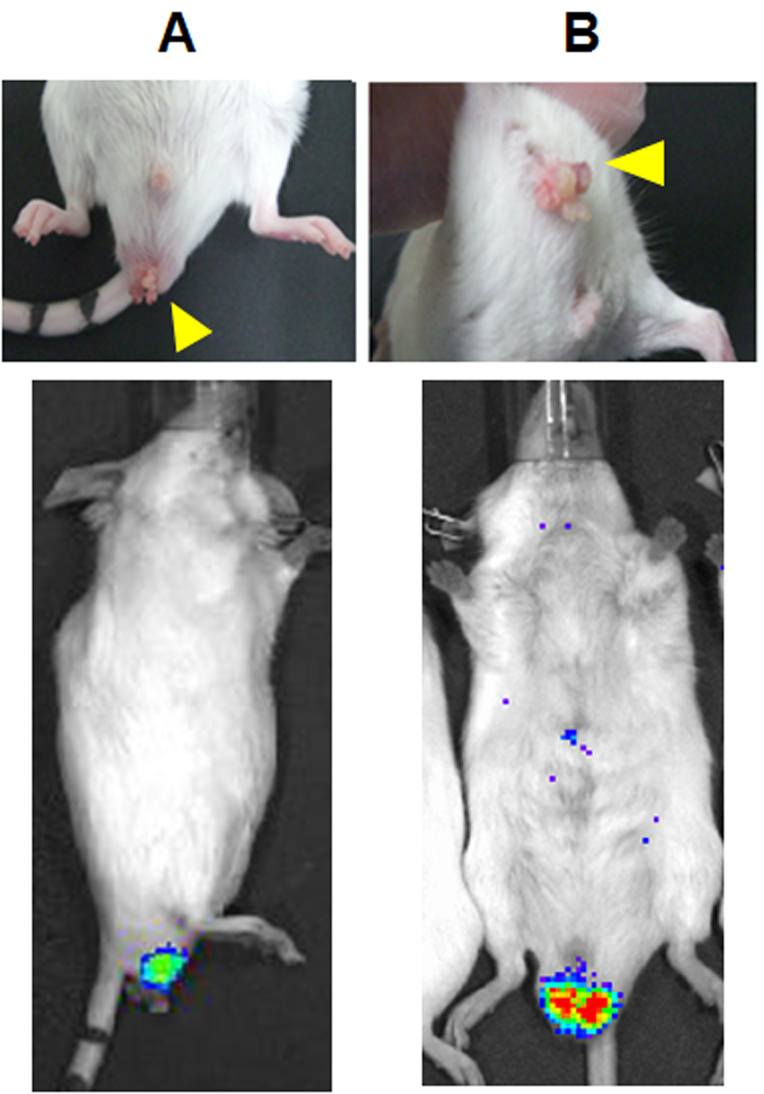

Supplement: Figure S7 — Images of papillomas. Photos of the papillomas in mice A and B were taken 18 weeks after the N-methyl-N-nitrosourea treatment. (TIF) [file pone.0026640.s007.tif]

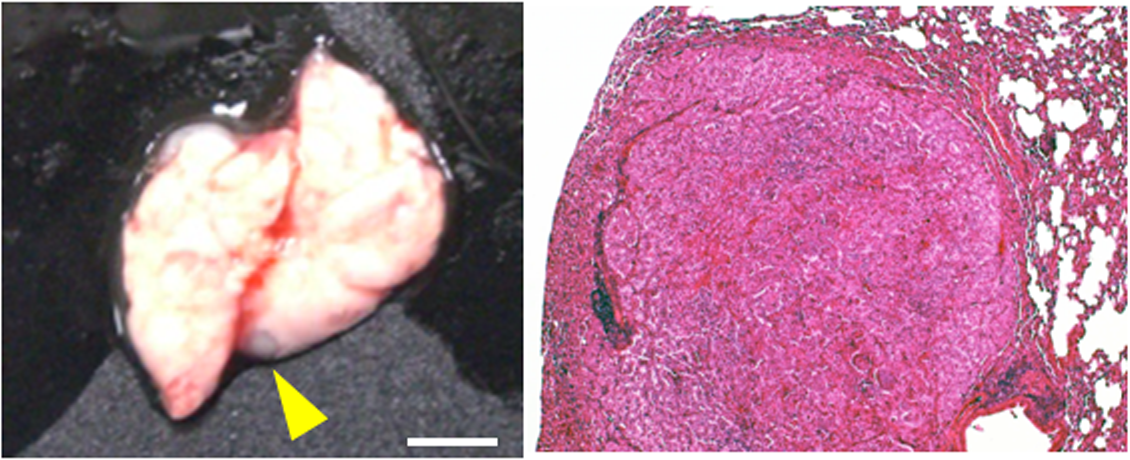

Supplement: Figure S8 — The alveolar/bronchiolar adenoma from mouse C. Bright field image of the lung (left) and tumor section (right, ×50) of the alveolar/bronchiolar adenoma (yellow arrow head in the left panel) from mouse C. Bar = 3 mm. (TIF) [file pone.0026640.s008.tif]
