# Supplementary material for: Detection of the Onset of Ischemia and Carcinogenesis by Hypoxia-Inducible Transcription Factor-Based In Vivo Bioluminescence Imaging
Source: PLoS One. 2011 Nov 10;6(11):e26640. doi: 10.1371/journal.pone.0026640 (PMC3213102; doi:10.1371/journal.pone.0026640)
Supplement: Text S1 — FISH analysis. (DOCX) [file pone.0026640.s009.docx]

Detection of the onset of ischemia and carcinogenesis by hypoxia-inducible transcription factor-based *in vivo* bioluminescence imaging

Tetsuya Kadonosono, Takahiro Kuchimaru, Shuichi Yamada, Yumi Takahashi, Atsushi Murakami, Hitomi Watanabe、Taeko Tani, Masahiro Inoue, Tetsuya Tsukamoto, Takeshi Toyoda, Tomoharu Tanaka, Kiichi Hirota, Koji Urano, Kazuhiko Machida, Tomoo Eto, Tomoyuki Ogura, Hideki Tsutsumi, Mamoru Ito, Masahiro Hiraoka, Gen Kondoh & Shinae Kizaka-Kondoh

**Supplementary methods**

**FISH analysis**

Spleen cells from ODD-Tg mice were cultured using Con A and LPS stimulation. Cells previously treated with thymidine were cultured with BrdU for R-banding, followed by colcemid incubation. After hypotonic swelling in 75 mM KCl buffer, cells were fixed and stored in methanol/acetic acid according to previously described methods [[1](#_ENREF_1)]. Before hybridization, cells were placed on slides and dried overnight. These were then washed with PBS and soaked in Hoechst 33258 for metaphase preparation followed by UV irradiation with a UV crosslinker (Stratagene, La Jolla, CA, USA). The probe DNA (the 2.2-kb transgene fragment) used was labeled with biotin-16-dUTP (Roche Applied Science, Penzberg, Germany) by nick translation. The probe was then mixed with sperm DNA and transfer RNA, and its precipitation was carried out with 100% ethanol. Denaturing was performed for 10 min at 75°C, and the probe was stored at 4°C until use. The hybridization steps of the chromosome preparation were performed as described previously [[2](#_ENREF_2)]. For detection of signals, avidin­­–fluorescein isothiocyanate (Roche Applied Science) was used. The chromosome preparations were stained for 30 min with propidium iodide. Observation of the chromosomes was performed with a Leica DM RXA2 fluorescent microscope (Leica Microsystems, Wetzlar, Germany). Metaphase chromosomes with signals were analyzed using CW4000-FISH (Leica).

**References**

1. Matsuda Y, Harada YN, Natsuume-Sakai S, Lee K, Shiomi T, et al. (1992) Location of the mouse complement factor H gene (cfh) by FISH analysis and replication R-banding. Cytogenet Cell Genet 61: 282-285.

2. Watanabe Y, Ebukuro M, Yagami K, Sugiyama F, Ishida J, et al. (1996) Chromosomal mapping of human angiotensinogen gene and human renin gene by fluorescence in situ hybridization (FISH) in transgenic mice. Exp Anim 45: 265-269.
